# Training Cameroonian researchers on pragmatic knowledge translation trials: a workshop report

**DOI:** 10.11604/pamj.2014.19.190.5492

**Published:** 2014-10-23

**Authors:** Lawrence Mbuagbaw, Lehana Thabane, Pierre Ongolo-Zogo

**Affiliations:** 1 Department of Clinical Epidemiology and Biostatistics, McMaster University, Hamilton, ON, Canada; 2 Biostatistics Unit, Father Sean O'Sullivan Research Centre, St Joseph's Healthcare-Hamilton, ON, Canada; 3 Centre for Development of Best Practices in Health, Yaoundé Central Hospital, Yaoundé, Cameroon; 4 Departments of Paediatrics and Anaesthesia, McMaster University, Hamilton, ON, Canada; 5 Centre for Evaluation of Medicine, St Joseph's Healthcare-Hamilton, ON, Canada; 6 Population Health Research Institute, Hamilton Health Sciences, Hamilton, ON, Canada; 7 Faculty of Medicine and Biomedical Sciences, University of Yaoundé 1, Yaoundé, Cameroon

**Keywords:** Pragmatic trials, knowledge translation, capacity building, Cameroon, workshop

## Abstract

Limited health research capacity in one of the factors that prevents developing countries from attaining optimal health outcomes and achieving the Millennium Development Goals. We report here, the details of a workshop on pragmatic knowledge translation trials for Cameroonian researchers, the material covered and additional resources to support capacity development. At the end of this workshop, knowledge gains were noted and participants were able to initiate proposals for funding. These proposals were aimed at improving the clinical management of diabetes, hypertension and malaria.

## Introduction

Many countries in Africa suffer from limited health research capacity [1, 2]. Ongoing efforts to improve health research capacity and promote evidence-based practice among Cameroonian clinicians and researchers have been met with many challenges. Key among these is the challenge of using or conducting evidence syntheses such as systematic reviews without basic understanding of primary study designs [3]. As such, efforts have been made to enhance capacity in primary research [4]. Randomized controlled trials are often considered to generate high quality evidence, but have been criticized for lacking generalizability [5]. Designing RCTs that generate findings that are readily applicable in “real world” settings require that they be pragmatic.

The United Nations’ Millennium Development Goals (MDGs) provide a framework for a comprehensive strategy to improve the lives of people in low-resource settings. The efforts described here address the fourth (reduce child mortality), sixth (combat HIV/AIDS and malaria) and eighth (global partnership for development) MDGs by focusing on the application of evidence for the management of morbid conditions through international partnerships. In a bid to enhance local capacity to generate context-relevant evidence that can readily be applied, building on information gathered from previous workshops [3, 4], and in response to research capacity needs, the Centre for Development of Best Practices in Health (CDBPH; www.cdbph.org) conducted a workshop on pragmatic knowledge translation trials. The CDBPH is a knowledge translation and brokering facility located in the Yaoundé Central Hospital, Yaoundé, Cameroon, that supports a wide range of health care stake-holders in the generation and application of research evidence. The CDBPH was created with funding from the Global Health Leadership Award from the Canadian Global Health Research Initiative administered by the International Development Research Centre (IDRC) – Canada.

This workshop sought to introduce the notion of “pragmatism” in evidence generation by guiding participants on research that bridges the evidence-practice gap [6]. The purpose of this report is to provide a detailed account of the activities, material and output of this workshop; and to showcase successful capacity building strategies.

### Workshop report

**Location:** The CDBPH organised the workshop, which took place from the 30^th^ June to the 2^nd^ August 2014, at the Laurence Vergne Room of the Yaoundé Central Hospital, Yaoundé, Cameroon.

**Aims:** The overarching theme of this training was to build on the efforts from previous workshops and to introduce Cameroonian clinicians and researchers to clinical trials [4]. This time the aim was to train participants on pragmatic knowledge translation trials and to develop a proposals for funding. Participants were expected to achieve the following competencies at the end of the workshop: 1) distinguish pragmatic trials from other types of trials, 2) understand key concepts in knowledge translation, 3) describe important steps in clinical trial design and 4) participate in the design of a pragmatic knowledge translation trial.

**Participants:** the CDBPH invited lecturers from the Faculty of Medicine and Biomedical Sciences, of the University of Yaoundé 1, staff from the Ministry of Health and other independent researchers affiliated with the CDBPH.

**Facilitators:** the course was facilitated by a Canadian professor of biostatistics and epidemiology with experience in pragmatic knowledge translation trials, a Cameroonian public health physician and epidemiologist; and a Cameroonian professor of radiology. All three facilitators had vast experience with knowledge translation, pragmatic trials and evidence-based practice.

**Pre-workshop tasks:** the pre-workshop tasks included introductory readings to knowledge translation and pragmatic trials, and to consider potential research questions that could be addressed during the workshop.

**Program:** Over two days, the participants were guided on the how to break down a research questions into its components using the PICOT framework (participants, intervention, comparison, outcome, timeframe); [7] and the basics of knowledge translation and pragmatic trials. Upon request, further details on clinical trial methodology were covered including sample size, unit of randomization, intervention, measurement, analyses, inference and reporting using the CONSORT (Consolidated Standards of Reporting Trials) statement [8]. They also covered the PRECIS (Pragmatic-Explanatory Continuum Indicator Summary) tool and practiced how to apply it to their own research [9]. The next two days involved hands-on group work and developing proposals for pragmatic knowledge translation trials. Among the topics brought up by the workshop participants (Table 1), three were chosen for development of detailed proposals. The participants, interventions, comparisons, outcomes and timelines are described in Table 2. Before and after the workshop a questionnaire was administered to test baseline and post-workshop knowledge of the material covered.

**Table 1:** Intervention research topics proposed by participants in knowledge translation pragmatic trials workshop

Topics
Telemedicine interventions to bridge human resources shortage
Checklists for improving malaria treatment guidelines
Community participation in public health programmes
Reducing acidosis to prevent end-stage kidney disease in dialysis patients
Mannitol before dialysis for reducing cerebral oedema
Efficacy of reminders to improving immunization coverage in children
Text messaging for preventing HIV infection in youth
Checklist for improving adherence to guidelines for treatment of hypertension
Honey as an oxytocic to prevent postpartum haemorrhage

**Table 2:** Outline of knowledge translation pragmatic trials in development

Components of trial design	Diabetes	Hypertension	Malaria
Participants	Medical doctors in health facilities	Medical doctors at the district level	Medical doctors and nurses in district hospitals and health centres
Interventions	**Main intervention:** 1. Memory aids **Add-on interventions:** 2. Reducing patient burden 3. Self-assessment 4. Continued medical education	**Main intervention:** 1. Poster guidelines with a decision aid algorithm **Add-on interventions:** 2. Training in workshops 3. Banning medical visitors 4. Reminders text messages 5. Increasing access to drugs in the guideline	**Main intervention:** · Checklist for adherence to malaria guidelines
Comparison	Current practice/guidelines only	Usual care	Standard care, guidelines, but no checklist
Outcome	**Practice outcomes:** Guidelines respected **Patient outcomes:** Diabetic control Mortality Morbidity **Process outcomes:** Duration of consultation	**Practice outcomes:** Adherence to guidelines **Patient outcomes:** Mortality Morbidity Blood pressure at 6 months	**Patient outcomes:** Number treated according to guidelines (exit interview and follow up **Patient outcomes:** Mortality, morbidity
Timeframe	Daily or weekly	After each consultation Six months duration	As per outcome

**Course material and readings:** the participants were provided with reading material relevant to each topic addressed. Table 3 is a summary of the topics covered in the workshop, the readings and other electronic resources.

**Table 3:** Workshop outline

Objective	Topics covered	Readings and Internet resources
1) Distinguish pragmatic trials from other types of trials	Clinical research and study design	Study designs: http://www.cebm.net/?o=1039; http://www.healthknowledge.org.uk/e-learning/epidemiology/practitioners/introduction-study-design-is-rct
	Unit of randomization, intervention, measurement and inference; sample size	[13–18]
	Pragmatic versus explanatory trials	[19, 20]
2) Understand key concepts in knowledge translation	Introduction to knowledge translation	Innovation to Implementation: A Practical Guide to Knowledge Translation in Health Care (Eng/Fr; http://www.sfu.ca/carmha/publications/i2i.html)
3) Describe the steps involved in conducting a clinical trial	Equipoise	[21]
	Research question formulation	[7] Asking focused questions: http://www.cebm.net/index.aspx?o=1036
	Overview of the steps involved in clinical trials	[22]
4) Participate in the design of a pragmatic knowledge translation trial	The PRECIS tool	[9, 19]
	The CONSORT extension for pragmatic trials	[23]
	Estimating the required sample size for a clinical trial	[24] Online sample size calculators: http://www.stat.ubc.ca/∼rollin/stats/ssize/ http://statpages.org/proppowr.html Free software for sample size estimation: http://www.brixtonhealth.com/pepi4windows.html
	Follow-up and attrition	[25, 26]
	Reporting a clinical trial	[27]
	Additional resources for clinical trials	Ethics: www.elearning.trree.org; www.tcps2core.ca General resources: www.globalhealthtrials.tghn.org Trial registration: www.pactr.org; www.clinicaltrials.gov Management of clinical trials: [28]

**Figure 1:**
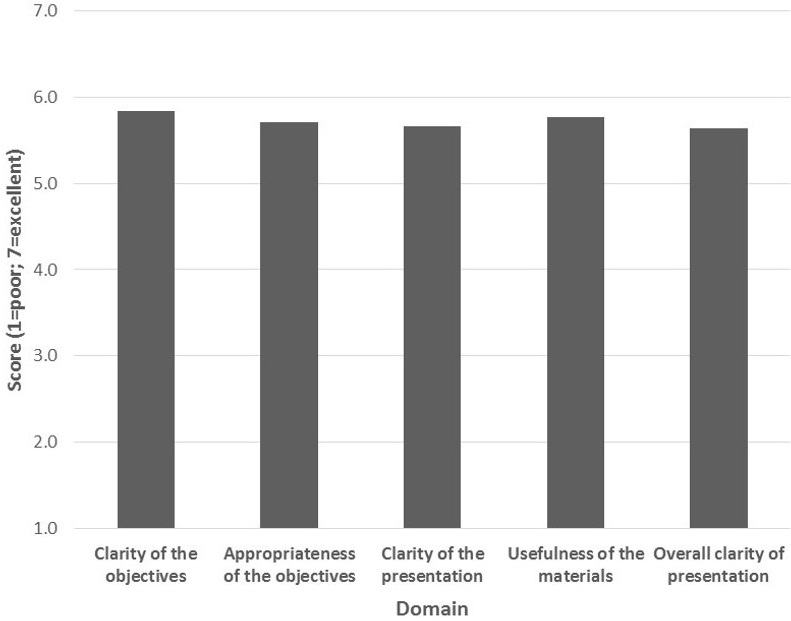
Evaluation of workshop (n=15)

**Evaluation:** The results of the questionnaire before and after the workshop were evaluated and compared. The questionnaire covered all topics discussed during the workshop. A total of 27 points could be obtained for responding correctly to 11 questions. The mean score (standard deviation) before the workshop was 14.7 (3.75) compared to 18.27 (4.21) after the workshop. This difference (+3.5) was statistically significant (t (33) =-2.64; p<0.012), showing a marked improvement in knowledge on pragmatic knowledge translation clinical trials. Participants were also asked to rank each of the sessions in the workshop in the following domains: Clarity of the objectives, appropriateness of the objectives, clarity of the presentation, usefulness of the materials and overall clarity of presentation. Overall, the workshop was well received (Figure 1). The workshop participants mostly liked the practical and “pragmatic” nature of the workshop, the group work sessions, availability of the facilitators, the material covered and detailed explanations provided during presentations. They wished the workshop could be longer and still had concerns regarding the PRECIS tool and some aspects of study design. They suggested the following improvements: more material on study design and knowledge translation; and more support for their personal research projects. For future workshops they wished to learn more about observational studies, sample size estimation, systematic reviews, critical appraisal and randomization. Upon request, the participants were given electronic copies of the material from previous workshops [4].

**Output:** Three draft proposals for knowledge translation pragmatic trials in the management of diabetes, hypertension and malaria were developed during this workshop. These three conditions contribute to significant morbidity and mortality in Cameroon. The prevalence of diabetes and hypertension are 6% and 24% respectively with close to 5 million Cameroonians affected by one or both of these conditions. Malaria on the other hand is even more morbid. It is responsible for close to 40% of all deaths in health facilities, 50% of all hospitalisations in children under 5 and 45% of all medical consultations [10]. These numbers suggest that the topics selected by the participants are highly relevant to Cameroon. In addition, recent papers suggest that management of malaria is sub-optimal with little or no respect for current national guidelines [11, 12]. These proposals are described in Table 2. Completion of these drafts and sourcing for funding is ongoing.

## Conclusion

Achieving the United Nations Millennium Development Goals (MDGs) which include curbing child deaths, reducing the morbidity and mortality from malaria (and HIV) and engaging in global partnerships will require not only financial investments by all countries, but commitment to strengthening health systems through capacity building in health research in developing countries. Building capacity in pragmatic trials to translate knowledge into practice is an imperative. In this paper, we report on the second in a series of workshops aiming at improving evidence-based practice in health by conducting pragmatic KT trials in Cameroon. The workshop generated several ideas where there is still a gap between knowledge and practice—management of patients with diabetes, hypertension and malaria. The proposals to develop these ideas into real trials are currently underway. The hope is that, if successfully funded, these will transform the healthcare system in Cameroon and hopefully spark the interest to conduct more KT trials led by African researchers in in sub-Saharan Africa—a region that remains the disproportionately affected by these diseases.
